# Complete Resumé of, Including Notices of Bills and Reports on Public Health, Subjects Issued by the Legislature during the past Quarter, and the Second Report of the Postmaster-General

**Published:** 1856-04

**Authors:** 


### SANITARY LEGISLATION.

### BILLS.

*Metropolis Local Management Act* (19 Vict., 22 February, 1856).—The Attorney and Solicitor-General have brought in a Bill to explain and amend the above-named Act. The bill declares that the expression "existing vestry," section 8 in the act, shall be construed to include all vestry meetings, meetings in the nature of vestry meetings, and meetings of the parishioners, inhabitants, or ratepayers generally; or of the parishioners, inhabitants or ratepayers rated at or above any specified amount or value, which might from time to time have been lawfully holden in the parish if the said act had not been passed. And from the time when, according to the said section 8, any "existing vestry" in a parish was to be superseded by the vestry constituted under the said act, all the duties, powers and privileges which might have been performed or exercised by an existing vestry, or any such meetings as aforesaid, shall be deemed to have become transferred to the vestry constituted by the above-named act, except so far as any duties, powers or privileges may, by section 90 of the act, be transferred to District Boards of Works. The bill further provides, that all duties and powers relating to the church, to the relief of the poor, or the administration of money or property for the poor, which at the time of the passing of the act were exercised by any commissioners, or any body other than the then existing vestry, shall continue to be exercised by such commissioners or other body as aforesaid.

*Medical Officers of Health.*—The greater number of the new appointments of Medical Officers of Health, under Sir B. Hall's new act, have been filled up; and in some instances the selections have been well made, in other cases the fortunate candidates are unknown in the sanitary world, and have yet to win their spurs in this field. There is abundant work before them; and such work as must show results, if it is well done. The mortality-tables of the Registrar-General will be a certain tell-tale of their exertions.

*Nuisances Removal Bill—Scotland* (19 Vict., February 26, 1856).—This Bill, brought in by the Lord-Advocate, provides for the repeal of preceding acts; places the superintendence of the execution of the act in the hands of the "General Prison Board," who may appoint medical inspectors; and entrusts the local administration to town councils, where they exist, or to the jurisdiction of police commissioners; or to the parochial board of any parish where the jurisdiction of a town council does not extend. The local authority is to have the right of appointing inspectors of nuisances and lodging-houses. The word "nuisances," under this bill, is widely defined. It implies (*a*) any insufficiency of size, defect of structure, want of repairs, or other matter rendering any inhabited building unwholesome or unfit for human habitation; (*b*) any pool, watercourse, ditch, gutter, drain, privy, urinal, cesspool or ashpit, so foul as to be injurious to health; (*c*) any animal so kept as to be injurious to health; (*d*) any accumulation or deposit, within the limits of any burgh, or within *fifty* yards of any dwelling-house, injurious to health; (*e*) any work, manufactory, trade or business injurious to the health of the neighbourhood. The bill also relates to the prevention or mitigation of diseases; to the regulation of common lodging-houses, and to the mode of enforcing the provisions of the proposed act.

*** The Bill is carefully drawn up; and although evidently based on very limited knowledge and views as to the true meaning of sanitary science, it would, in the event of its becoming law, do some certain amount of service.

*Burial Grounds* (*Ireland*) *Bill* (1856), to amend the laws relating to the burial of the dead in Ireland. The Bill proposes to vest the management of burials in burial boards, and gives the Lord-Lieutenant power to restrain the opening of new burial places, and to order discontinuance of burials in specified places; and orders that every coffin shall be covered by at least fifty-four inches of earth.

*Bill to consolidate and amend the Acts relating to Vaccination* (1856).—The new Vaccination Bill is brought in by Mr. Cowper and Mr. Bouverie. It proposes to place vaccination under the local control of boards of guardians; a step, as we take it, at once fatal to success, even though such boards be placed under the superintendence of the Board of Health. Any legally qualified medical man may be appointed vaccinator, 2*s*. 6*d*. being the lowest fee for the operation. The bill is based on the compulsory system. Apropos to this bill, we have before us two interesting papers on state vaccination, from Dr. E. T. Hughes and Dr. Spencer Thomson. Both are in favour of compulsory vaccination; but Dr. Thomson suggests that all persons should have the privilege of selecting their own qualified medical man as vaccinator, and that such medical man should, within six months after birth, be allowed to select the time for the operation.

*Bill to encourage the providing of Improved Dwellings for the Labouring Classes in Ireland* (19 Vict., February 14, 1856).—This Bill, prepared by Sir W. Somerville and Mr. Hamilton, provides for the erection of tenements for the Irish poor, possessing certain requisites, such as stone walls, at least two separate rooms, a sufficient external window which can be opened for ventilation, a sufficient chimney built of stone, a sufficient privy, a separate pigstye, and an open space, 18 feet wide, before the dwelling.

---

### REPORTS AND RETURNS.

*Second Report of the Postmaster-General on the Post-Office* (30 January, 1856).—The Duke of Argyll, as Postmaster-General, has brought out a very interesting report. At page 31 his Grace refers to the unhealthy condition of the homes of the letter-carriers, and makes the important suggestion of erecting model houses for them, near the General Post-Office, with moderate rents. Such a plan would save to the men time and trouble, and would be convenient to the department. It is proposed that such dwellings should be put up by a public company, rather than by government.

At pages 74-6, the report of the medical officer (Dr. Waller Lewis) occurs, and is highly satisfactory. By the present regulation, the medical officer has charge of 1,071 officers, and partial charge of 387 more, whom, in times of epidemic suffering, he visits at their own homes. During such times, also, he extends his supervision to 295 gentlemen with higher salaries, and to the officers of the departments generally. The chief diseases which Dr. Lewis has met with amongst the postmen are, autumnal diarrhœa and rheumatism. Out of 986 cases of disease in total, 76 were cases of rheumatism. No other diseases can be said to have prevailed extensively. About 50 per cent. of the officers under Dr. Lewis's care have received medical aid during the six months just elapsed. The deaths amount to nine, one in a clerk (consumption); eight amongst letter-carriers, three from consumption (ages under 30); one from hypertrophy of the heart (age between 50 and 60); one from dropsy (age between 50 and 60); and one from softening of the brain (age 30).

Of 300 candidates examined for office, 21 were rejected, from physical incapacity. Among the removable causes of disease, Dr. Lewis names "draughts of cold air from the doors of the Inland Circulation and Newspaper Office; escape of contaminated air from the men's water-closets into different parts of the buildings; inefficient ventilation in the passages, lobbies, bag-rooms, and messengers' kitchens." The clerk of the works is now carrying out a plan for remedying these evils—some of which are already removed. The buildings appear to be perfectly dry.

Dr. Lewis, in conclusion, remarks on the insanitary condition of the private homes of the letter-carriers, and refers also to the propriety of erecting model houses for them, near the office, as some of them have to walk several miles in addition to the distances required by their duties.

*District Lunatic Asylums in Ireland.* (February 5th, 1856.)—A very interesting return, drawn up by Messrs. Donaldson and Wilkes, commissioners. It refers to the new district asylums, twelve in number. The statements of the commissioners, we regret to say, are unfavourable, as regards some of the more important sanitary requirements of these asylums. Owing to the extent of the corridors, an equable temperature cannot be sustained. The ventilation of the bedrooms is attempted in various ways, but in none effectively; they are close and offensive at night. The supply of water is derived from wells which, in many instances, have failed: the employment of patients to pump up the water is attended with ill consequences. Drainage is not super-excellent. The furniture fittings are scanty, and the bedsteads unsafe and totally unfit for an asylum.

*The St. Pancras Workhouse.* (1856.)—Dr. Bence Jones having been commissioned to inquire into the state of the St. Pancras Workhouse, has drawn up his report. It discloses a spectacle truly revolting. Whatever could be found in the way of filth, overcrowding, impure air, and such like disease-factors, Dr. Jones found in luxurious development.

The following are the author's remedial suggestions:—"The out-door relieving offices should be removed at once. Immediate steps also should be taken to lessen the crowded state of the house, by entirely removing the schools; for this, amongst other reasons, that an epidemic of low fever is at the present moment in the parish, and among the inmates, as is shown by the fact that during the first four weeks of this month (1st to 29th), forty-five patients have been attacked by fever. The arrangement by which the casual women are mixed with the women waiting for admission into the workhouse in the receiving ward is exactly that which is most liable to introduce and keep up an outbreak of low fever. These wards should be immediately separated. It is very desirable that an entirely new building should be made for an infirmary, where the access of foul air would be prevented, and a perfect system of ventilation could be adopted. Meanwhile, if the only good building at present existing might be used as the infirmary, many of the immediate difficulties of the house would be obviated."

*State of the Army in the Crimea.* (1856.)—The report of Sir John M'Neil and Colonel Tulloch on the disasters of the Crimean army, while it fully supports the statements made by the press, presents numerous facts of grave interest, in a sanitary point of view. We must seize another opportunity to extract from this able and honest document the sanitary lessons which it teaches.

*Mortality Registration in Scotland: Over-Legislation.*—We are informed by our excellent fellow-labourer, Dr. John Webster, that the registration of births, deaths, and marriages, throughout Scotland, promises great results, supplies a desideratum long felt, and removes the opprobrium that North Britain was almost the only country in Europe where such registration was unknown. Still the measure is as yet defective, and, in one special point, highly objectionable; viz., that it obliges any medical practitioner, under a penalty of forty shillings, who shall have been in attendance during the last illness until the decease of any patient, to transmit within fourteen days after death a certificate of such death to the registrar. This proceeding is most arbitrary, and has justly given great umbrage to professional men. If legislators consider penalties advisable in such cases, they should place the responsibilities on the relatives of the deceased—certainly not on the medical attendant, who perhaps may only casually have seen the patient. What renders the compulsory clause more objectionable is, that no remuneration is provided for the medical attendant who is forced to supply the information.

When recently visiting Scotland, Dr. Webster met with various medical friends, who complained seriously of this grievance, and stated that they had actually been compelled under penalty to certify to the causes of the deaths of individuals to whom they had only given casual advice, and respecting whose disease and its subsequent fatal termination they retained scarcely any recollection. This is over-legislation, with a vengeance; and is not only unjust, but is sufficient to prevent anything like accuracy in the returns.

